# Targeted Thiazole Orange Derivative with Folate: Synthesis, Fluorescence and *in Vivo* Fluorescence Imaging 

**DOI:** 10.3390/molecules15106983

**Published:** 2010-10-11

**Authors:** Xuening Fei, Yingchun Gu, Yiqi Wang, Qingyang Meng, Baolian Zhang

**Affiliations:** 1 School of Chemical Engineering and Technology, Tianjin University, Tianjin, 300072, China; Email: guyingchun@tjuci.edu.cn (Y.-C.G.); 2 Department of Material Science and Engineering, Tianjin Institute of Urban Construction, Tianjin, 300384, China; Email: yiqi3778@163.com (Y.-Q.W.); dream12114@163.com (Q.-Y.M.); ybysw@126.com (B.-L.Z.)

**Keywords:** thiazole orange, folate, synthesis, targeted label *in vivo*, fluorescence imaging

## Abstract

A Thiazole Orange conjugated with folate derivative was synthesized in two steps. Firstly, folate was coupled with 1-(3-aminopropyl)-4-methylquinolinium bromide to afford folate-methylquinolinium bromide, which then reacted with benzothiazolium to obtain the title folate-conjugated compound. The compound was evaluated by ^1^H-NMR MS, TG/DTA and fluorescence spectroscopic methods. The title compound could selectively target folate receptor expressing tumors according to the *in vivo* fluorescence imaging preliminarily performed on nude mice with breast tumors.

## 1. Introduction

An increasing scientific and commercial interest in the synthesis and application as probes of cyanine dyes, whose spectra can reach near-infrared region, has aroused much attention in recent years. These probes can be used as powerful detection or treatment tools in biological systems [1,2,3,4,5,6,7,8,9], such as tools allowing detection of DNA and RNA in gel by flow cytometry or microscopy. Embedded fluorescent dyes such as Thiazole Orange (TO) and Oxazole Yellow (YO) are of particular interest because they have lower quantum yields and weaker fluorescence in solution, while larger fluorescence enhancements are observed when bound to DNA or RNA partners [10]. When the dye is inserted into a DNA molecule, especially the double-helix of DNA, the fluorescence intensity can be enhanced more than 1,000 times, and more than 3,000 times when inserted into RNA. This kind of dyes has higher affinity towards tumors than to the normal cells, a feature that can be widely used in the early-stage labeling of cancer cells, as the obvious distinction of fluorescence between free dye and nucleic acid-bound dye provides an excellent way to image, label and detect cancer. 

Folate receptors (FR), which are 38 kDa glycosylphosphotidylinositolanchored proteins, exist in three major forms, namely, FR-α, FR-β, and FR-γ. The FR-α form is over-expressed in various types of human carcinomas, including ovarian, endometrial, breast, renal cell carcinomas, and so forth [11,12,13]. It is displays high affinity for the vitamin folic acid. The fact that high-affinity for FR is retained when folate is covalently linked via its γ-carboxyl group to a foreign molecule [14,15,16] makes folate a valuable vehicle for conjugation with specific tracers, allowing the delivery of the tracers to FR-positive cancer cells and providing cancer specific imaging. Successful tumor selective FR-targeting has been reported. Kennedy *et al.* [17] used the tumor targeting ligand folic acid to deliver an attached fluorescent probe to tumors overexpressed by the folate receptor. Upon laser excitation, derived images of normal tissues generally show little or no fluorescence, whereas images of folate receptor-expressing tumors display bright fluorescence that can be easily distinguished from adjacent normal tissue. The sharp distinction between tumor and normal tissues provided by this technique could find application in the localization and resection of tumor tissue during surgery or in the enhanced endoscopic detection and staging of cancers. Bunz *et al.* [18] synthesized folate-PPE as a fluorescent contrast agent to image cancer cells. The fluorescent polymer targeted and imaged KB cancer cells *in vitro* with high selectivity, which was evidenced by laser scanning confocal microscopy and fluorescence microscopy. 

**Scheme 1:**
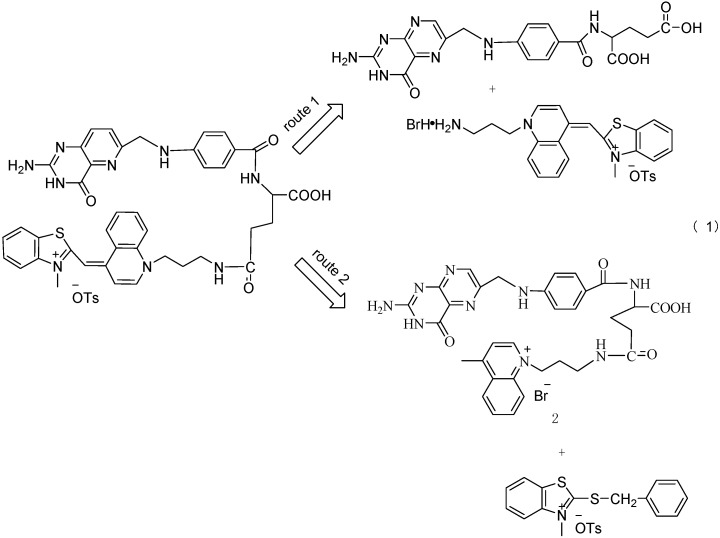
Retrosynthesis of folate-TO-NH_2_.

We expected that folic acid conjugated with TO could selectively deliver the organic fluorescence probe to folate-receptor-overexpressing breast cancer cells and thus utilize TO’s advantages. In our recent studies, we obtained TO derivatives by both liquid and solid phase synthetic methods via introduction of substitutent groups on the benzothizaole and quinoline rings. The TO derivatives were then used to label living cells and satisfying results were obtained [19,20]. In this paper, for targeting purposes, the cyanine dye TO with an amine residue (TO-NH_2_) was modified by folic acid to obtain a coupled folate-TO (Route 1 in Scheme 1), which was applied to targets to facilitate the identification of cancer cells with extra FR on the membrane cellular surface. It is not so easy to synthesize or modify folate-TO because the amine residue is not so stable and may be oxidized. This fact motivated us to prepare TO-folate dye through another way (Route 2 in Scheme 1).

**Scheme 2:**
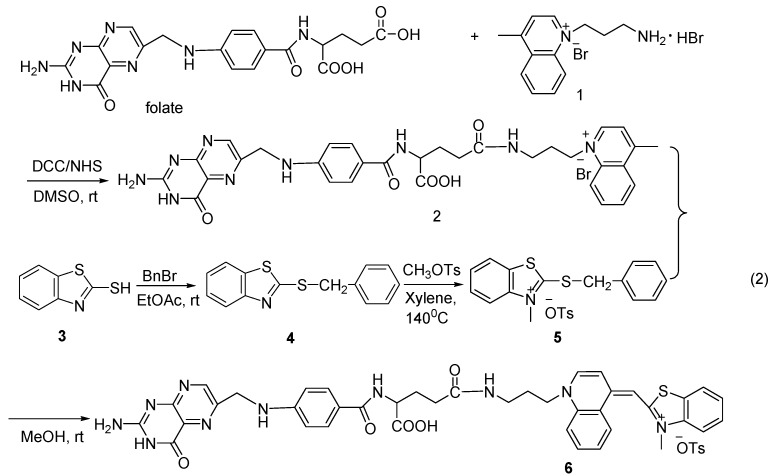
Synthesis of folate-TO-NH_2_.

In this paper, we have designed and synthesized a conjugated probe based on folate and TO. Folate was used as a protecting group for the primary amine of 1-(3-aminopropyl)-4-methylquinolinium bromide, after which the deprotection was unnecessary and then the protected compound reacted with benzothiazolium to obtain the folate-TO directly. The details for the syntheses are shown in Scheme 2.

## 2. Results and Discussion

### 2.1. Synthesis

The method for preparation of this folate-TO involves two essential steps: (1) conjugation of folate with 1-(3-aminopropyl)-4-methylquinolinium bromide (**1**) to afford folate-quinolinium bromide (**2**); (2) reaction of the folate-quinolinium bromide with 2-methylthio-*N*-methylbenzothiazolium tosylate (**5**) to obtain folate-TO. 

Starting from compound 2-mercaptobenzothiazole (**3**), folate compound **2** was synthesized and then reacted with compound **5** to obtain compound **6**. Folate compound **2** can be obtained from the reaction of folate and compound **1**. Briefly, folate was activated using two equivalents each of DCC and NHS and then reacted with two equivalents of 1-(3-aminopropyl)-4-methylquinolinium bromide at room temperature to give compound **2**. 

Folate compound **2** can also be synthesized from NHS-folate and compound **1** in DMSO. First, folate was activated by NHS to obtain NHS-folate, and then two equivalents of 1-(3-aminopropyl)-4-methylquinolinium bromide were added at room temperature. Compound **1** was derived from 4-methylquinoline. Compound **5** was synthesized by quaterization of compound **4**, which was obtained by alkylation of compound **3** with benzyl bromide. Finally, compound **6** was prepared after the reaction of compounds **2** and **5** in DMSO for 48 h.

### 2.2. Thermal analysis

The results were further strengthened by differential thermal analysis. The sample was scanned at a rate of 5 °C min^−1^ from 30°C to 700 °C. There were endothermic peaks at 156 °C, 302 °C and 420 °C, respectively, while there was an exothermic peak at 454 °C on the DTA curve of compound **1**. Meanwhile, an 80% weight loss was observed within 280–340 °C, meaning that compound **1** had decomposed. There were endothermic peaks at 156 °C and 423.4 °C, respectively, and an exothermic peak at 442 °C on the curve of folate. On the curve of folate compound **2**, endothermic peaks at 151.3 °C and 431.9 °C and exothermic peaks at 170.5 °C and 448.6 °C were observed, respectively. This indicated that the folate compound curve has similar features to that of folate **2**. Differences and relations between the three DTA thermograms suggested that folate and compound **1** were conjugated to afford compound **2**. An endothermic peak at 148.6 °C on the curve of folate compound **6** was similar to that of compound **5** at 158.3 °C, while on the other hand, two new endothermic peaks at 402.2 °C and 472.3 °C and a large exothermic peak at 454.8 °C resembled those of compound **2**. According to the three curves, a conclusion was drawn that folate compound **2** and compound **5** were conjugated to afford folate compound **6**.

### 2.3. NMR spectra

NMR spectral chemical shifts of folate-conjugated TO further confirmed the conjugation. The signals of folate backbone and compound **1** were found on the spectra of folate compound **2**, while those of folate backbone and TO in that of compound **6**. There was no peak at 12.36 ppm corresponding to carboxyl group on the spectra of folate compound **2** and **6**, compared to that of folate, which indicated that γ-carboxyl group of folate was conjugated with the amine residue.

### 2.4. Fluorescence and ultraviolet properties

**Figure 1:**
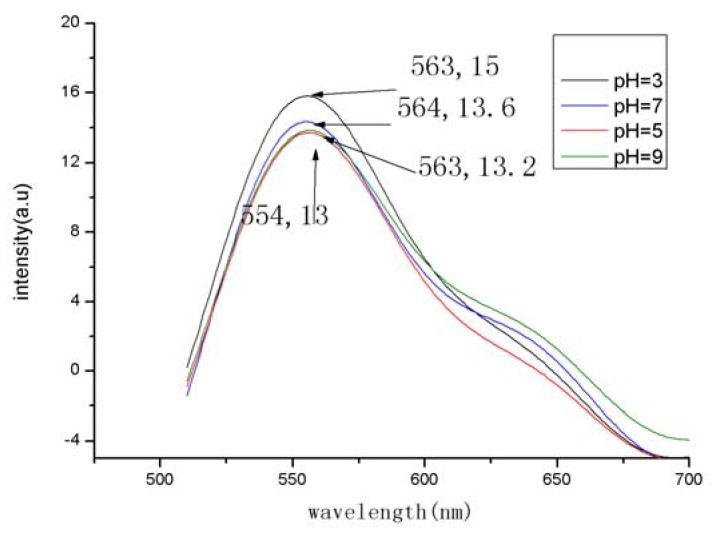
Fluorescence spectra of folate-TO at different pH (excition at 480 nm was used with slit width of 10 nm and emission slit width of 10 nm).

The fluorescence intensities under different pH conditions were measured with a Cary Eclipse fluorescence spectrophotometer at a fixed excitation wavelength of 480 nm with a slit width of 10 nm. The fluorescence emission spectrum of folate-TO-NH_2_ presented a red-shift of 13 nm bearing a characteristic fluorescence spectrum resembling that of TO-NH_2_ in DMSO. While the fluorescence emission wavelengths of the title compound appeared no shift at different pH with only a little change of the fluorescence intensity (Figure 1).

**Figure 2:**
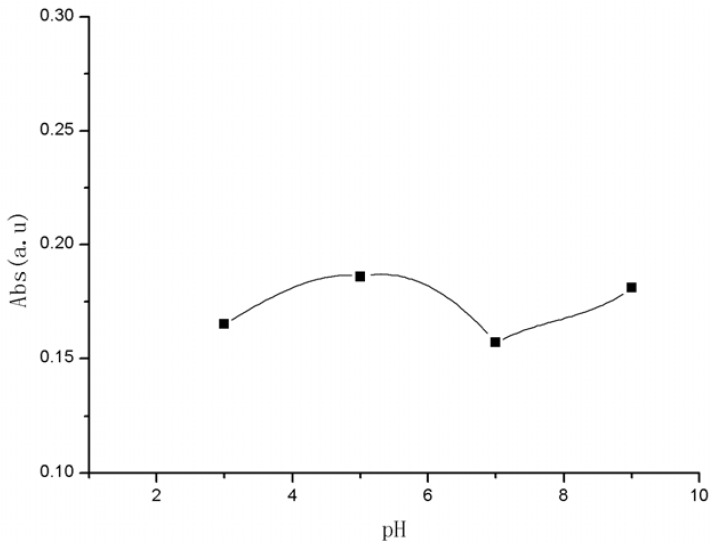
UV absorption spectra of folate-TO at different pH.

In the ultraviolet spectrum (Figure 2)., the absorption peak of folate-TO was at 510 nm, which was similar to that of TO-NH_2_, showing that compound 2 was bound with compound **5**. The absorption peak changed little while the absorption intensity was about 0.18 with a little change under various pH values (3–9). 

### 2.5. Fluorescence image in vivo

**Figure 3:**
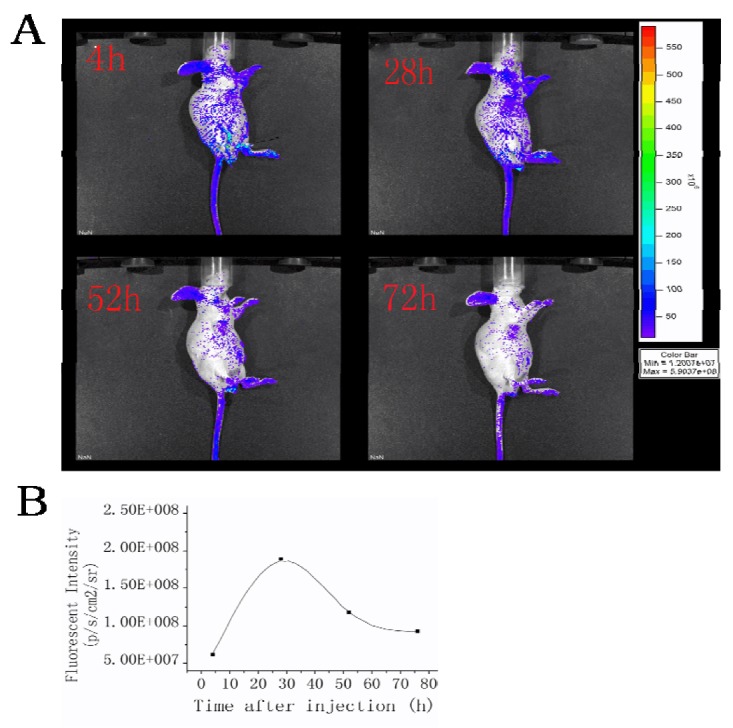
(**A**) Fluorescence images of MFC-7 tumor mice in real time postinjection of folate-TO. (**B**) Fluorescent intensities at different injection times.

In order to confirm the targeting effect of folate-TO, fluorescent imaging based on the specific marking of tumors was carried out. MFC-7 cells were cultured. For imaging, five female 6–8 week old nude mice were anesthetized with 1.5-2% isoflurane in oxygen administered via a facemask. Then 20 μL of the suspended MFC-7 cell was inoculated into the breast tissues of each mouse. Fluorescent probe of folate-TO was administered to mice with cancer cells via tail vein injection and the fluorescence imaging was performed before and during the experiment at various times after the injection of folate-TO. The mice became fluorescent, and the tumor could be clearly delineated from the surrounding background tissue after injection in 4 h (Figure 3A). The fluorescence intensity was measured by photons per second per square centimeter per steradian (p/s/cm2/sr) in the tumor as a function of time, which is depicted in Figure 3B.

This indicates that immediately after the injection folate-TO was rapidly and systemically distributed throughout the mice, including the tumor xenografts, as shown in Figure 3. After 28 h, folate-TO fluorescence regions clearly defined the zone of the cancer cells. The subsequent results indicated a decrease of fluorescence intensity, possibly due to a redistribution of the fluorescing probe. After 52 h the diffuse systemic fluorescence pattern began to recede, and the regions of cancer were clearly defined. Fluorescence signals were still emitted from the breast tumor at 72 h postinjection. 

Our results proved that folate-TO is able to penetrate tumors and target FR expressing ones *in vivo* stably and selectively. The nude mice were alive at 6 d postinjection. The folic acid conjugated compound can enter the intracellular cytoplasm via endocytosis and selectively deliver the organic fluorescence probe to breast cancer cells to label them.

## 3. Experimental

### 3.1. General

Fluorescence spectra were scanned on a Cary Eclipse fluorescence analysis instrument (American). Mass spectral analyses were obtained using an electrospray ionization (ESI) mass spectrometer of LCQ Advantage (Thermo Fisher, American). Melting points were taken on a Yanaco apparatus and were uncorrected. ^1^H-NMR spectra were recorded on a Bruker AC-P300 (300 MHz) spectrometer. Chemical shifts are reported in parts per million (ppm) downfield from TMS (tetramethylsilane). DTA thermograms analysis was carried out using a Perkin–Elmer simultaneous thermo-gravimetric/differential thermal (TG/DTA) analyzer. UV absorption spectra were recorded on a T6 visible spectrophotometer (China). All the reagents were analytically pure.

### 3.2. Biology

#### 3.2.1. Cell lines

MFC-7 cell line was bought from Shanghai Queen & King Biochem Co,.LTD. In the cell culture hood, a sterile glass or plastic pipet was used to transfer the contents of the vial slowly into the tube containing the growth medium. The vial of cells was transfered to a 37 °C water bath until the suspension was just thawed. MFC-7 cells were cultured in Dulbecco’s modified Eagle´s medium (DMEM) supplemented with 10% (v/v) of FBS. All cells were grown at 37 °C in a humidified atmosphere containing 5% CO_2_ [21]. Cells should be subcultured when they reached 80% confluence and digested with 0.25% trypsin. After trypsinization, DMEM medium was used to collect and deposit the cells at 1000 r/min for 10-minute centrifugation. After that, the cells were transplanted to a 24-well plate and the cell concentration was about 1 × 10^5^ /mL for each well. Synchronous cells were obtained by the serum deprivation for 24h. The tissues were put on precleaned microscope slides (Fisher Scientific) and covered with another microscope slide. 

#### 3.2.2. Fluorescent imaging *in vivo* and biodistribution studies

For imaging, a female 6–8 week old nude BALB/c mouse was used. Before the experiment, the anaesthetized animal was fixed on the supporting plate of the container. During the experiment, the animal was placed vertically in a container consisting of a supporting plate and a covering glass plate that was slightly pressed to fix the animal. The distance between the plates was about 1 cm. Then 20 μL of the cell suspension MFC-7 was inoculated into the breast tissue of the mouse. Fluorescent probe folate-TO was administered to mouse via tail vein injection at a concentration of 7.5 μM. *In vivo* fluorescence imaging was performed before and various times following the injection of folate-TO-NH_2_ with a Xenogen IVIS200 Lumina imaging system (Xenogen, USA).

### 3.3. Preparation of folate- 1-(3-amidepropyl)-4-methylquinoline bromide *(**2**)*

Folic acid (1 equivalent) and triethylamine (2 equivalents) were dissolved in DMSO (10 mL) in a 50 mL flask under a flow of nitrogen with magnetic stirring. Dicyclohexylcarbodiimide (DCC) (2 equivalents) and NHS (2 equivalents) were added to the flask. The solution was stirred for 1 h at room temperature in the dark and compound **1** was added. The mixture was stirred overnight in the dark at room temperature, filtered via glass wool to remove the insoluble dicyclohexylurea byproduct, and precipitated using cold acetone and diethyl ether. Compound 2 was collected by filtration, washed and dried under vacuum. Yield: 92 % ^1^H-NMR (DMSO-*d*_6_): δ 1.89–2.10 (m, 4H, 2H of folate), 2.32 (t, *J* = 7.80 Hz, 2H, folate) 2.73 (s, 3H), 2.93 (t, *J* = 5.10 Hz, 2H), 4.31–4.35 (m, 1H, folate), 4.48 (s, 2H, folate), 5.04 (t, *J* = 6.15 Hz, 2H), 6.64(d, *J* = 8.70 Hz, 2H, folate ), 6.96 (t, *J* = 5.70 Hz, 1H, CH_2_NH of folate), 7.65 (d, *J* = 8.70Hz, 2H, folate), 7.85–7.95 (m, 2H), 8.04 (t, *J* = 7.05 Hz, 1H), 8.13 (d, *J* = 7.2 Hz, 1H, -CONH of folate), 8.27 (d, *J* = 8.40 Hz, 1H), 8.56 (d, *J* = 7.20 Hz, 1H), 8.65 (s, 1H, folate), 9.34 (d, *J* = 6.90 Hz, 1H), 11.52 (s, 1, H, folate-OH); ESI-MS: 624.3 (M^+^), 625.3 (M^+^ + 1).

### 3.4. Preparation of folate-TO *(**6**)*

Compound **2** (1 equivalent) was dissolved in DMSO (30 mL). Compound 5 (2 mL, 2 equivalents) dissolved in CH_3_OH were then added. The reaction mixture was stirred at room temperature in the dark for 24 h and precipitated by cold acetone and diethyl ether. The precipitate was collected and washed with acetone, diethyl ether and water. Yield: 86% ^1^H-NMR (DMSO-*d*_6_): δ 1.87–1.90 (m, 1H, folate), 1.99–2.01 (m, 1H, folate), 2.28–2.32 (m, 4H, 2H of folate), 3.14–3.18 (m, 2H), 3.98 (s, 3H), 4.30–4.37 (m, 1H, folate), 4.48 (d, *J* = 5.70 Hz, 2H, folate), 4.75 (t, *J* = 6.15 Hz, 2H), 6.63 (d, *J* = 8.70 Hz, 2H, folate), 6.84 (s, 1H), 6.97 (d, *J* = 8.70Hz, 1H, CH_2_NH of folate), 7.05–7.12 (m, 2H), 7.19 (d, *J* = 7.20 Hz, 1H), 7.37–7.50 (m, 2H), 7.64 (d, *J* = 8.70 Hz, 2H, folate), 7.75(d, *J* = 8.40Hz, 1H) 7.92–8.00 (m, 2H), 8.16 (d, 1H, *J* = 6.6Hz, -CONH of folate), 8.55 (d, *J* = 7.20 Hz, 1H), 8.63 (s, 1H, folate), 8.74 (d, *J* = 8.40 Hz, 1H), 11.48 (s, 1H, folate-OH); ESI-MS: 771.4 (M^+^), 772.6 (M^+ ^+ 1), 773.7 (M^+^ + 2).

## 4. Conclusions

In conclusion, we have reported a convenient synthetic method to obtain a folate conjugated probe (folate-TO) .The reactions are easy to carry out and the starting materials are readily available. The preliminary fluorescence imaging of mice was studied and the results show that folate-TO can be used to selectively penetrate and label tumors for further study on the information about cell nucleus.
